# Analysis of factors influencing the efficacy of ovarian vein embolization for pelvic venous insufficiency and development of a short-term efficacy prediction model with internal validation

**DOI:** 10.1016/j.jvsv.2025.102441

**Published:** 2026-01-05

**Authors:** Binyu Zheng, Yangzheng Xia, Ranting Ye, Dongmei Liu, Gaorui Liu, Yong Liu

**Affiliations:** a Department of Ultrasound, Beijing Shijitan Hospital, Beijing, China; b Western Sydney Vascular, Westmead, New South Wales, Australia

**Keywords:** Ovarian vein embolization, Pelvic venous insufficiency, Minimal clinically important difference, Short-term treatment efficacy, Predictive model

## Abstract

**Objective:**

The study aims to elucidate the factors associated with the short-term efficacy of ovarian vein embolization (OVE) in the treatment of pelvic venous insufficiency (PVI), to construct a predictive model for short-term efficacy of OVE.

**Methods:**

Clinical and ultrasound data were retrospectively collected from female patients with PVI and underwent OVE at Beijing Shijitan Hospital between January 2019 and February 2025. This study used the minimal clinically important difference to define symptomatic improvement in the Pelvic Venous Clinical Severity Score. Symptomatic improvement was used as the evaluation criterion, and the related factors affecting the short-term efficacy were analyzed. The receiver operating characteristic curve was also applied to assess the predictive efficacy of the model and calculate the optimal efficiency cut-off value. Internal validation was performed using k-fold cross-validation to assess discrimination, calibration, and clinical utility.

**Results:**

The study included a total of 82 patients: 43 patients in the remission group and 39 patients in the nonremission group. No statistically significant differences were found between the two groups with regard to age, body mass index, history of abortion, history of varicose veins of the lower extremities, the number of pregnancies and deliveries. The duration of lower abdominal discomfort in the nonremission group was longer than that in the remission group (*t* = −1.713; *P* = .004; independent samples *t* test). Transabdominal ultrasound showed that the diameter of the left ovarian vein (OV) in the nonremission group was smaller (*Z* = −2.958; *P* = .003; Mann-Whitney *U* test), and the diameter of the left parametrial vein in the nonremission group was smaller (*Z* = −2.494; *P* = .013). In addition, the positive rate of internal iliac vein reflux in the nonremission group was higher (χ^2^ = 15.649; *P* < .001; χ^2^ test or Fisher's exact test). Binary logistic regression analysis showed that the longer the duration of lower abdominal discomfort (odds ratio [OR], 1.115; 95% confidence interval [CI], 1.001-1.332; *P* = .049), the smaller the diameter of the left OV (OR, 0.669; 95% CI,0.483-0.928; *P* = .016) and internal iliac vein reflux (OR, 6.449; 95% CI, 2.238-15.583; *P* < .001) were independent risk factors for the short-term efficacy of OVE for PVI. The area under the receiver operating characteristic curve (AUC) was 0.807 (95% CI, 0.712-0.902), and the best cut-off value was 0.453. The sensitivity and specificity of predicting the short-term efficacy of OVE were 82.1% and 74.4%, respectively. Internal validation showed acceptable discrimination (area under the receiver operating characteristic curve = 0.779), a Brier score of 0.176 indicating adequate accuracy, reasonable calibration, and positive net clinical benefit in decision curve analysis.

**Conclusions:**

The duration of lower abdominal discomfort, OV diameter, and internal iliac vein reflux are independent predictors of the short-term efficacy of OVE for PVI. The prediction model of short-term efficacy of OVE for PVI in this study has satisfactory validity.

**Clinical Relevance:**

This study developed an exploratory predictive model to evaluate the risk of nonremission at 3 months after OVE in patients with symptomatic PVI. Three key predictors were identified: duration of pelvic discomfort, left OV diameter, and the presence of internal iliac vein reflux. The model enables preoperative identification of patients at high risk for adverse short-term outcomes.

**Article Highlights**

• **Type of Research:** Single-center retrospective cohort study.
• **Key Findings:** Short-term symptomatic remission after ovarian vein embolization for pelvic venous insufficiency was observed in 52.4% of patients at 3 months, based on a clinically meaningful improvement in symptom severity. Poor short-term outcomes were associated with a longer duration of pelvic pain, smaller left ovarian vein diameter, and the presence of internal iliac vein reflux. A prediction model incorporating these factors showed good discrimination and acceptable internal validation.
• **Take Home Message:** Clinically meaningful symptom improvement provides a practical framework for evaluating early outcomes after ovarian vein embolization. Preoperative symptom burden and pelvic venous hemodynamics, particularly internal iliac vein reflux, play a central role in determining short-term treatment response and may help to guide patient counseling and follow-up strategies.

Chronic pelvic pain refers to nonperiodic pain in the lower abdomen or pelvis lasting >6 months. Frequently associated with pelvic venous disorder, this condition presents with a complex etiology, challenging diagnosis, and suboptimal treatment efficacy, imposing a significant physical and psychological burden on patients.^1^ Pelvic venous insufficiency (PVI) is considered as one of the most important causes of chronic pelvic pain. Primary venous reflux or secondary obstruction involving the iliac vein, ovarian vein (OV), renal vein, or inferior vena cava may constitute the underlying causes of PVI. Patients typically present with abdominal pain, lower back pain, dyspareunia, menorrhagia, and vaginal discharge. Effective PVI treatments include medication, open surgery (eg, hysterectomy, salpingectomy, or oophorectomy), and interventional procedures. Among these, OV embolization (OVE) combined with foam sclerotherapy is regarded as both effective and safe. Long-term follow-up (>1 year) has demonstrated remission of symptoms in >80% of patients.^2^^,^^3^ However, symptoms can persist in some patients postoperatively. Most patients endure prolonged pain before PVI diagnosis and harbor high expectations for OVE short-term efficacy. This study retrospectively analyzed clinical and ultrasound data from PVI patients with and without symptom remission at 3 months post OVE. It identified the risk factors associated with short-term nonremission, with particular focus on the early nonremission subgroup, and developed a predictive model to estimate the likelihood of short-term symptoms nonremission after OVE. Our overarching objective was to support preoperative clinical evaluation and provide a framework for the long-term management of patients experiencing after the procedure.

## Methods

### Patient recruitment

Retrospective collection of clinical and ultrasound data from 82 female patients with PVI at our hospital between January 2019 and February 2025. Inclusion criteria included the following: (1) aged ≥18 years old; (2) presence of nonmenstrual, chronic, or recurrent lower abdominal/pelvic pain, or other symptoms related to pelvic venous disorder; (3) both duplex ultrasound examination and digital subtraction angiography (DSA) confirming the presence of PVI: OV reflux and unilateral or bilateral parauterine and perivaginal vein diameters of ≥4.0 mm; (4) has undergone OVE; and (5) completion of the Pelvic Venous Clinical Severity Score (PVCSS). Exclusion criteria included the following: (1) patient was diagnosed with gynecological disease(s), including endometriosis, adenomyosis, pelvic inflammatory diseases, pelvic adhesions, congenital developmental abnormalities, and/or tumors; (2) patients have gastrointestinal, urological, spinal disorders, pubic neuralgia, or myofascial pain; (3) previous gynecological or pelvic surgeries; (4) history of thrombosis of the inferior vena cava and iliac veins; (5) with portal hypertension; and (6) menstruation, pregnancy, and 1-year post delivery.

The study was approved by the local ethical committee of the Beijing Shijitan Hospital, Capital Medical University (protocol No. IIT2024-016-003) and registered at Chinese Clinical Trial Registry (ChiCTR2400080369). All participants were informed during their examination that their data might be used for research purposes and provided written informed consent.

### Evaluation criteria for the treatment efficacy of OVE

The PVCSS was used to comprehensively evaluate clinical symptoms of PVI patients at two key time points: before OVE procedure and at 3 months after surgery.^4^^,^^5^ Additionally, multiple methods were used to determine the minimal clinically important difference (MCID) value of PVCSS, and postoperative patients were divided into two groups: (1) remission group: the difference of PVCSS between preoperative and postoperative of ≥4.5 points; (2) nonremission group: the difference between preoperative and postoperative changes in PVCSS was <4.5 points.

### MCID calculation method

#### Anchor-based method

The anchoring method mainly compares changes in quality of life with an external standard.^5^ In general, by evaluating the correlation between the anchor and the target scale, the correlation coefficient value of ≥0.3 is considered to be relevant, and the anchor can be used to determine the MCID.^6^ This study used the patient global impression of change (PGIC) scale as the anchor.^7^ To verify the applicability of the PGIC score as an anchor, Spearman rank correlation analysis was used to evaluate its correlation with the difference in preoperative and postoperative PVCSS scores of the patient. As shown in Table I, a strong correlation between the two groups was found (ρ = −0.870; *P* < .01), indicating that the PGIC is a valid anchor measure.

**Table I:** Spearman's correlation between Pelvic Venous Clinical Severity Score (*PVCSS*) score change and patient global impression of change (*PGIC*) anchor

Variable	Spearman's Rho	95% CI	*P* value
PVCSS change vs PGIC score	−0.870	−0.915 to −0.804	<.001

*CI,* Confidence interval.

N = 83 for all correlations.

**Fig 1:**
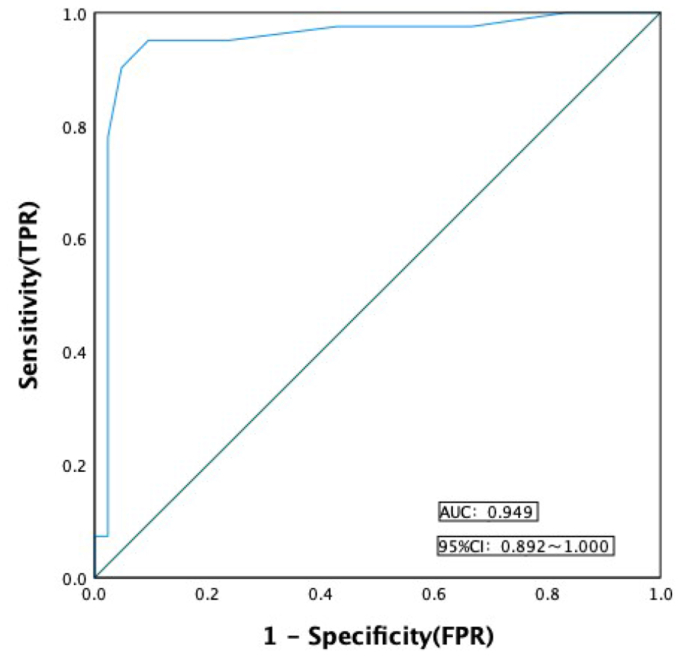
The receiver operating characteristic (ROC) curve of the consistency between the changes in Pelvic Venous Clinical Severity Score (PVCSS) score and the improvement of clinical symptoms on the patient global impression of change (PGIC) scale. *FPR*, false-positive rate.

The PGIC is a 7-point scale, with higher scores indicating worse patient self-rated treatment, with a score of 1 representing complete disappearance of symptoms, a score of 4 representing no significant change in symptoms, and a score of 7 representing significant deterioration in symptoms. Consistent with prior studies using PGIC dichotomization, we defined scores of 1 to 3 as remission and 4 to 7 as nonremission.^8^ Patients were divided into remission and nonremission groups. Receiver operating characteristic (ROC) curve analysis was used to evaluate the consistency of PGIC score with the difference of PVCSS score before and after surgery. Fig 1 shows that a decrease in PVCSS score of ≥4.5 points after the OVE procedure could be used as the optimal cut-off value to distinguish nonremission from remission (area under the receiver operating characteristic curve [AUC] = 0.949; 95% CI, 0.892-1.000).

#### Distribution-based method

The distribution method mainly relies on the statistical distribution of baseline scores of the scale.^6^ In this study, the MCID was calculated based on the standard deviation (SD) of the score change of PVCSS, with the MCID = 0.5 × SD baseline. According to the distribution method, patients with a decrease of PVCSS score of <2.9 (0.5 × 5.818) before and after surgery were classified as the remission group, whereas those with PVCSS score decrease of ≥2.9 were categorized as the nonremission group.

In this study, the cut-off value determined by the anchor-based method (4.5 points) was higher than the MCID estimated using the distribution-based approach (2.9 points). As highlighted by Jayadevappa et al,^9^ nearly one-half of existing research uses both methods to establish MCID values, emphasizing their significance in scale development. Because the anchor-based approach relies on patient-reported outcomes, it is considered more clinically relevant and thus prioritized. Although a 2.9-point change may be statistically significant, a greater decrease is generally necessary to capture a meaningful subjective improvement. Therefore, this study defined clinical remission after OVE as a decrease of ≥4.5 points in PVCSS scores, with patients scoring below this threshold categorized as nonremission.

### Factors that may affect the efficacy of OVE

**Fig 2:**
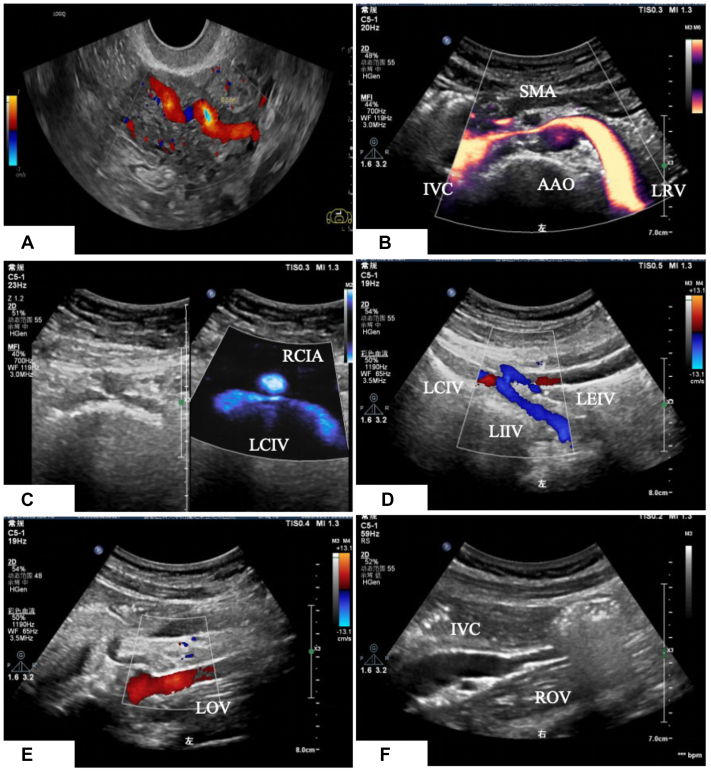
Ultrasound images of the abdominopelvic vessels. **(A)** Arc-shaped uterine veins with a left-to-right shunt. **(B)** Nutcracker phenomenon. **(C)** Left common iliac vein compression. **(D)** Left internal iliac vein reflux. **(E)** LOV reflux. **(F)** ROV. *AAO*, abdominal aorta; *IVC*, inferior vena cava; *LEIV*, Left external iliac vein; *LIIV*, left internal iliac vein; *LOV*, left ovarian vein; *LRV*, left renal vein; *RCIA*, right common iliac artery; *ROV*, right ovarian vein; *SMA*, superior mesenteric artery.

The dataset comprises two categories of variables. (1) Clinical data include age at surgery, duration of abdominal discomfort, number of pregnancies, number of deliveries, history of abortion, history of lower extremity varicose veins, and body mass index. (2) Ultrasound data include the diameter of bilateral uterine and paravaginal veins, diameter of bilateral OVs and reflux pattern (spontaneous, intermittent or induced), presence of left iliac vein compression, nutcracker phenomenon and internal iliac vein reflux (Fig 2).

### The surgical methods of OVE

After antegrade puncture of the left femoral vein with an 18G needle and placement of a 6F short sheath, angiography was performed to visualize the pelvic venous system, including the iliac veins, OVs, renal veins, and inferior vena cava. Under the guidance of DSA, the distal end of the main OV was identified. Subsequently, two to three controllable coils (15 × 400 mm; PocoInterlock) were deployed at the proximal and distal end of the OV, and a total of 5 mL 3% foam sclerosing agent was injected between the coil and the distal end of the OV. Postprocedural DSA confirmed successful embolization, with no residual reflux observed in the OV.

### Statistical methods

Analysis of factors related to therapeutic efficacy: The data were analyzed using the IBM SPSS®23 software package with a two-sided test and an α of 0.05. A statistically significant difference was considered when the *P* value was <.05. For continuous variables, the Kolmogorov-Smirnov normality test was used first. Variables that conformed to normal distribution were expressed as mean ± SD and independent samples *t* tests were used to compare the two groups. Variables that did not conform to a normal distribution were expressed as median (quartile 1, quartile 3). Comparisons between the two groups were made using the Wilcoxon rank-sum test. Comparisons between groups of categorical variables were analyzed using the χ^2^ test or Fisher's exact test.

### Model construction

To identify risk factors associated with short-term nonremission of PVI after OVE, binary logistic regression analysis was conducted. Significant predictors were incorporated into a regression-based predictive model, with the calculated probability of postoperative nonremission serving as the test variable. The model's performance was assessed by generating a ROC curve.

#### Performance assessment and cut-off optimization

The ROC curve was analyzed to evaluate the discriminatory power of the model. The AUC was interpreted as follows.

• AUC < 0.70: low predictive efficiency.
• AUC = 0.70 to 0.90: moderate efficiency.
• AUC > 0.90: high efficiency.

The optimal cut-off point for prediction was determined using the Youden index, defined as: Youden index = sensitivity + specificity − 1. The cut-off value corresponding with the maximum Youden index was selected to achieve optimal balance between sensitivity and specificity.

#### Nomogram and internal validation

A nomogram was constructed based on the final binary logistic regression model to provide a user-friendly tool for estimating the probability of short-term nonremission after OVE. To evaluate model robustness, repeated k-fold cross-validation was applied. Internal validation was performed using repeated k-fold cross-validation. Model performance was evaluated in AUC, Brier score, Hosmer-Lemeshow test, and decision curve analysis.

**Fig 3:**
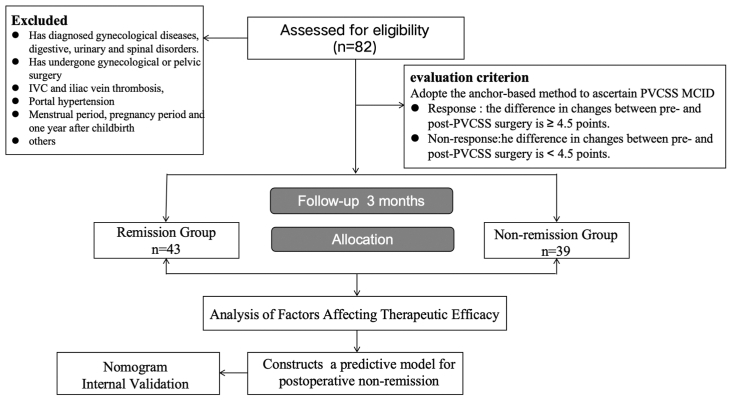
The research road map. *IVC*, inferior vena cava; *MCID*, minimal clinically important difference; *PVCSS*, Pelvic Venous Clinical Severity Score.

Binary logistic regression analyses were performed using IBM SPSS23 software package. The nomogram was developed and internal validation with k-fold cross-validation was conducted using R (version 4.2.3). Fig 3 provide the research road map.

## Results

A total of 135 patients were initially reviewed, of whom 82 patients diagnosed with PVI met the inclusion criteria and were included in the analysis. After OVE, 43 patients (52.4%) demonstrated short-term clinical remission and were assigned to the remission group. The remaining 39 patients (47.6%) did not achieve remission and were categorized as the nonremission group. Among nonremission cases, the mean preoperative PVCSS was 17, and the postoperative PVCSS was 15, indicating only marginal symptomatic improvement despite intervention. Based on these results, the related factors of short-term remission group and nonremission group were analyzed respectively.

### Difference analysis between OVE treatment of PVI and clinical data

The duration of lower abdominal discomfort in the nonremission group was longer than that in the remission group (*t* = −1.713; *P* = .004), although there was no significant difference in age, number of deliveries or pregnancies, history of abortion, and history of lower extremity varicose veins between the two groups (*P* > .05).

### Difference analysis between the efficacy of OVE in the treatment of PVI and ultrasound imaging data

There were statistically significant differences between the two groups in the diameter of the left OV (Z = −2.958; *P* = .003), the diameter of the left parametrial vein (Z = −2.494; *P* = .013), and the presence of internal iliac vein reflux (χ^2^ = 15.649; *P* < .001). In addition, the presence or absence of Nutcracker phenomenon, internal iliac vein and left iliac vein compression, left OV reflux pattern, right OV diameter, and right uterine vein diameter had no statistically significant impact on the short-term remission rate after OVE treatment (Table II).

**Table II:** Comparison of general clinical data and ultrasound data of the two groups of patients

Characteristics	Relief group (n = 43)	Nonrelief group (n = 39)	Statistic	*P* value
Age, years	41.4 ± 11.1	41.6 ± 12.6	−0.047^a^	.639
Age at surgery, years	40.9 ± 11.0	41.3 ± 12.4	−0.163^a^	.588
Duration of pain, years	3.1 ± 2.9	4.7 ± 5.3	−1.713^a^	**.004**
Body mass index, kg/m^2^	21.3 (19.7-22.8)	21.5 (19.8-23.8)	−0.599^b^	.549
No. of pregnancies	2.4 ± 1.3	2.4 ± 1.3	−0.051^a^	.529
No. of births	1.6 ± 0.8	1.4 ± 0.8	0.832^a^	.325
Combined miscarriage	58.1	69.2	1.084^c^	.298
Combined varicose veins	16.3	7.7	1.408^c^	.235
Combined nutcracker phenomenon	55.8	43.6	1.222^c^	.269
Left OV diameter, mm	7.9 (6.8-9.6)	6.6 (5.8-8.1)	−2.958^b^	**.003**
Spontaneous reflux	79.1	79.5	0.564^c^	.754
Combined left common iliac vein compression ≥50%	39.5	41.0	0.019^c^	.891
Combined left internal iliac vein incompetence	23.3	66.7	15.649^c^	**<.001**
Left uterine and paravaginal vein diameter, mm	6.9 (6.5-8.6)	6.2 (5.3-7.5)	−2.494^b^	**.013**
Right uterine and paravaginal vein diameter, mm	5.9 (5.3-6.5)	6.2 (5.1-7.1)	−0.795^b^	.427

*OV,* Ovarian vein.

Data are presented as number (%), mean ± standard deviation, or median (interquartile range).

Boldface *P* values indicate statistical significance.

a Independent sample *t* test.

b Wilcoxon rank-sum test.

c χ^2^ test or Fisher's exact test.

### Selection of predictive factors and construction of the predictive model

Binary logistic regression analysis showed that the duration of lower abdominal discomfort, the diameter of left OV, and internal iliac vein reflux were independent predictors of the short-term efficacy of OVE for PVI. The diameter of the left OV was a protective factor for the efficacy of OVE in the treatment of PVI (odds ratio [OR], 0.669; 95% confidence interval [CI], 0.483-0.928; *P* = .016).The duration of lower abdominal discomfort (OR, 1.115; 95% CI, 1.001-1.332; *P* = .049), and internal iliac vein reflux (OR, 6.449; 95% CI, 2.238-15.583; *P* < .001) were the risk factors of this study. The longer the duration of lower abdominal discomfort, the narrower the diameter of the left OV, or presence of internal iliac vein reflux, the lower the short-term remission rate of OVE treatment (Table III).

**Table III:** Logistic regression analysis of risk factors for short-term nonresponse patient after ovarian vein embolization (OVE) treatment for pelvic venous congestion syndrome

						95% CI	
Characteristics	β value	SE value	Wald value	*P* value^a^	OR value	Lower limit	Upper limit
Constant	1.542	1.263	1.491	.222			
Duration of pain, years	0.144	0.073	3.887	.049	1.155	1.001	1.332
LOV diameter, mm	-0.402	0.167	5.800	.016	0.669	0.483	0.928
Left IIV incompetence (1 = yes; 0 = no)	1.864	0.540	11.915	<.001	6.449	2.238	18.583

*β,* Regression coefficient; *CI,* confidence interval; *IIV,* internal iliac vein; *LOV,* left ovarian vein; *OR,* odds ratio; *SE,* standard error.

a Logistic regression analysis.

**Fig 4:**
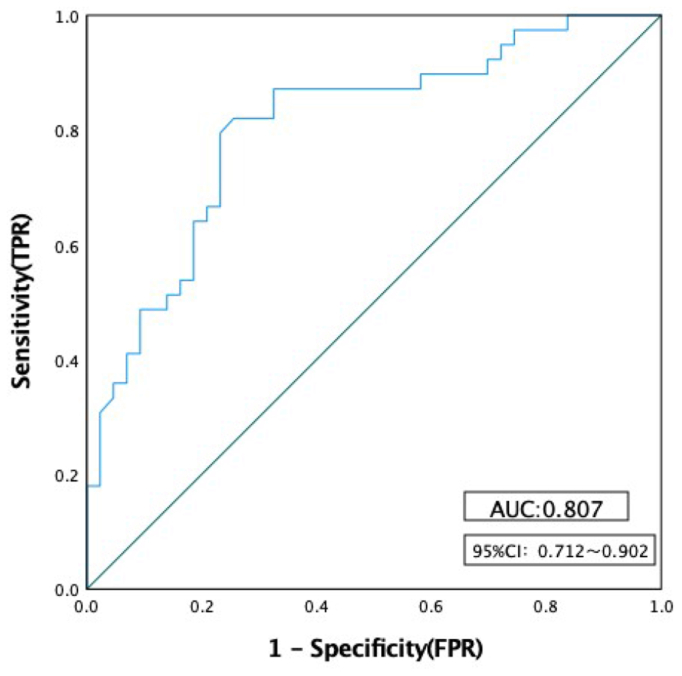
The receiver operating characteristic (ROC) curve of the predictive model for short-term efficacy nonresponse in ovarian vein embolization (OVE) treatment of pelvic venous congestion syndrome. *AUC*, area under the curve; *CI*, confidence interval; *FPR*, false-positive rate; *TPR*, true-positive rate.

The duration of pain, the diameter of the left OV, and the presence of internal iliac vein reflux were used as independent predictors. The prediction probability of nonremission of symptoms at 3 months after OVE (nonremission rate) was used as the test variable (remission = 0, nonremission = 1), and the prediction model was successfully constructed. At the same time, an ROC curve was drawn, and the best cut-off value of the model was 0.453, and the area under the ROC curve (AUC) was 0.807 (95% CI, 0.712-0.902) (Fig 4).

### Model performance

**Fig 5:**
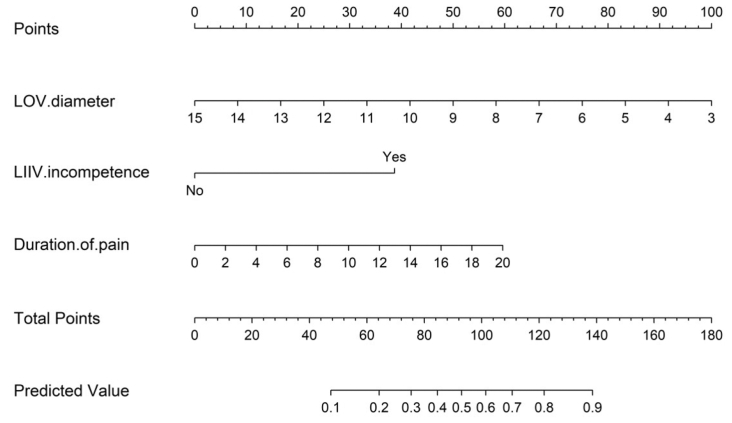
Nomogram predicting the probability of short-term (3-month) efficacy after ovarian vein embolization (OVE) in patients with pelvic venous insufficiency (PVI). *LIIV*, left internal iliac vein; *LOV*, left ovarian vein.

Internal validation results showed that the model had good discrimination (AUC = 0.779; 95% CI, 0.734-0.825). The Brier score was 0.176, indicating acceptable overall predictive accuracy. The calibration intercept (−0.098) and slope (1.00) were close to the ideal values, suggesting good overall calibration. The Hosmer-Lemeshow test was significant (*P* < .05), reflecting statistical deviation, but this test is sensitive to sample size and grouping; in conjunction with the calibration plot, the overall calibration was still considered acceptable. Decision curve analysis further demonstrated a positive net clinical benefit across a wide range of threshold probabilities. A nomogram based on the final predictive model is presented in Fig 5.

## Discussion

This study retrospectively analyzed symptom improvement of 82 patients with PVI at 3 months after OVE treatment. The short-term remission rate after OVE was 52.4%, and the nonremission rate was 47.6%. This study compared clinical and ultrasound data between patients who encountered remission and those who did not at the 3-month follow-up. It was found that the remission group experienced a shorter duration of pain compared with the nonremission group. Additionally, the inner diameters of the left OV and left parametrial vein were larger in the remission group, whereas the nonremission group exhibited a greater proportion of patients with internal iliac vein reflux. The duration of lower abdominal discomfort, diameter of the left OV, and presence of internal iliac vein reflux were found to be closely associated with short-term symptomatic remission after OVE for PVI.

Previous studies reported long-term remission rates (mostly >1 year) after OVE treatment ranging from 68% to 100%.^3^^,^^10^^,^^11^ Our study focused on patients who did not experience significant remission of symptoms in the short term after OVE treatment, with a short-term remission rate of 52.4%, which aligns well with findings from previous studies.^12^ Although most studies have used the visual analogue scale as a subjective quantitative measure of pain to evaluate the efficacy of OVE surgery, the present study used the PVCSS scale to evaluate treatment outcomes in PVI patients. The PVCSS comprises 10 clinical manifestations and signs, each scored on a scale of 0 to 3 based on subjective and objective severity. A higher total score indicates more severe disease. Most studies have shown that the PGIC can be effectively used by patients to self-evaluate overall changes or disease severity before and after treatment, especially in the context of chronic pain disorders or neurological function recovery.^7^ The MCID can promote the clinical application of quality-of-life scales and serves as a key parameter to evaluate the effectiveness of clinical decision-making.^13^

A comprehensive review of the literature revealed that there is currently no standardized criterion for assessing the clinical efficacy of OVE worldwide. Moreover, although statistical analysis has validated the PVCSS as an effective tool for evaluating surgical outcomes in PVI patients,^4^ there is no established MCID cut-off value to objectively distinguish whether symptoms have improved post OVE. As such, this study introduced the use of the PGIC scale as an anchor to formulate the MCID for the PVCSS, aiming to provide a more objective and clinically relevant benchmark for assessing the therapeutic efficacy of OVE.

The mechanism of pain caused by PVI remains unclear, although some studies suggest that it is related to hemodynamic changes and neurobiological factors.^14^ In this study, we observed that prolonged pain duration is a risk factor affecting symptomatic remission after OVE. Chronic pelvic venous dilatation and inflammation in PVI patients can increase the levels of neuropeptides such as calcitonin gene-related peptide and substance P, leading to peripheral and central nerve sensitization. This process increases the nerve pain threshold and contributes to residual pain.^15^ Furthermore, some patients continued to experience pain and discomfort after OVE owing to long-term spinal flexion caused by persistent lower abdominal discomfort, with spinal muscle damage requiring longer recovery times (beyond the short-term postoperative period).

In addition, a particularly important finding of this study is that internal iliac vein reflux is a risk factor for short-term symptomatic remission following OVE. Possible explanations include that (1) the internal iliac vein reflux is recognized as an important secondary cause of PVI.^16^ Ultrasound examination has shown no significant correlation between internal iliac vein reflux and iliac vein compression, and iliac vein compression does not necessarily lead to internal iliac vein reflux.^17^ (2) The pelvic venous system is complex, with collateral circulation easily forming between regional veins. The iliac vein plays a key role in connecting the abdomen and pelvis. The OV is connected to branches of the internal iliac vein through the uterovaginal venous plexus. The internal iliac vein lacks venous valves, making it prone to reflux when venous pressure increases.^18^ Therefore, internal iliac vein reflux can be secondary to OV reflux and acts as an overflow point for OV reflux.^19^ After OVE treatment, cessation of reflux in the OV may lead to increased abdominal venous pressure. This not only exacerbates venous reflux in patients with preexisting internal iliac vein reflux in the short term, but also further promotes the establishment of pelvic collateral circulation, ultimately leading to insufficient remission of symptoms after the intervention. Interestingly, we found that some patients who did not achieve short-term remission after OVE had significant improvement in symptoms during long-term follow-up (>6 months), accompanied by the resolution of internal iliac vein reflux upon ultrasound reexamination. Therefore, regarding the management of patients with OV reflux combined with internal iliac vein reflux, based on our findings and the latest guidelines, we recommend prioritizing OVE in the treatment of PVI.^20^ For patients whose symptoms persist postoperatively, regular imaging follow-up is essential to monitor hemodynamic changes in reflux veins. Further intervention may be warranted for patients exhibiting persistent venous reflux and minimal symptomatic improvement after OVE. Given that many individuals with PVI experience chronic psychological distress, including long-standing anxiety and depression, and often maintain elevated expectations for procedural outcomes, clinicians should prioritize comprehensive preoperative counseling. This approach is particularly critical in cases involving complex venous etiologies, such as coexisting internal iliac vein reflux, where the efficacy of OVE as a standalone intervention may be limited.

Jambon et al^21^ previously showed that a smaller diameter of the left OV (<7 mm) was associated with better outcomes after OVE. In contrast with their findings, we found that a larger left OV diameter correlated with improved short-term efficacy of OVE. In our study, all 82 patients showed dilation with reflux in the left OV on both ultrasound and DSA examinations. Specifically, the mean diameter of the left OV was approximately 8.15 mm in the remission group, compared with approximately 6.96 mm in the nonremission group. However, there was no obvious dilatation and reflux of the right OV. The possible reasons for this discrepancy could be as follows. (1) In Jambon et al's study, 79.8% of patients underwent both OVE and internal iliac vein embolization, whereas the current study mainly focused on the predictive factors of the efficacy in patients undergoing left-sided OVE alone. (2) The nonremission group in our study may have had a greater proportion of internal iliac vein reflux, which leads to a more complex etiology of PVI. In these cases, ovarian venous reflux might not be the dominant factor in the etiology of PVI, thereby decreasing the effectiveness of OVE. However, our study did not stratify patients based on the presence of OV reflux alone vs combined ovarian and internal iliac venous reflux. Further research is needed to evaluate symptom remission in patients with multifactorial PVI after embolization. (3) Last, a larger diameter of OV may indicate more obvious reflux, making the diagnosis more definitive, the indication for intervention stronger.

Although OVE has been proved to be effective for PVI and offers favorable long-term outcomes, previous studies have reported that 0% to 40% of patients experience no significant symptom remission or even symptom aggravation postoperatively.^19^ Apart from neurological factors, the formation of new collateral vessels or persistent internal iliac vein reflux post procedure may account for persistent symptoms in some patients. This study is the first to use the PVCSS scale to classify patients based on remission status after OVE. In addition, it focuses on the clinical and sonographic data of patients with short-term nonremission after OVE, providing a basis for the long-term management and postoperative evaluation of PVI.

### Limitations

The relatively small sample size and the single-center, retrospective design of this study may limit the generalizability of the findings. The predictive model proposed still needs external validation; using broader, multicenter datasets is essential to confirm the predictive performance of the proposed model, and expansion of the dataset is planned for future research. Additionally, the study emphasizes short-term postoperative outcomes, without incorporating long-term follow-up data. This factor restricts the ability to characterize the medium- and long-term clinical course and imaging features of patients who fail to achieve remission after OVE.

## Conclusions

Ultrasonography effectively evaluates pelvic venous abnormalities in PVI. This study was the first to apply the MCID to define symptomatic improvement in the PVCSS. Our exploratory study found that longer pain duration, narrower left OV diameter, and internal iliac vein reflux are independent predictors of poor short-term outcomes after OVE. The nomogram based on these factors provides a valuable tool for preoperative assessment, and the internal validation indicated that the model was well-calibrated. With further validation in larger, independent cohorts, this approach has the potential to support personalized treatment planning, guide targeted postoperative surveillance, and improve patient outcomes.

## Author contributions

Conception and design: BY, YZ, RT, DM, GL, YL

Analysis and interpretation: BY, YZ, RT, DM, GL, YL

Data collection: BY, YZ, RT, YL

Writing the article: BY, DM, GL, YL

Critical revision of the article: BY, YZ, RT, DM, GL, YL

Final approval of the article: BY, YZ, RT, DM, GL, YL

Statistical analysis: BY, GL, YL

Obtained funding: Not applicable

Overall responsibility: YL

## Funding

This research project received grants from China State Railway Group Co. Ltd (J2023Z604) and Capital's Funds for Health Improvement and Research (2024-2-2085). However, there was no involvement in any aspect of the research process.

## Disclosures

None.

## References

[1] Lamvu G., Carrillo J., Ouyang C., Rapkin A. (2021). Chronic pelvic pain in women: a review. JAMA.

[2] Senechal Q., Echegut P., Bravetti M. (2021). Endovascular treatment of pelvic congestion syndrome: visual analog scale follow-up. Front Cardiovasc Med.

[3] Hanna J., Bruinsma J., Temperley H.C. (2024). Efficacy of embolotherapy for the treatment of pelvic congestion syndrome: a systematic review. Ir J Med Sci.

[4] Akhmetzianov R.V., Bredikhin R.A. (2021). Clinical efficacy of conservative treatment with micronized purified flavonoid fraction in female patients with pelvic congestion syndrome. Pain Ther.

[5] Akhmetzyanov R.V., Bredikhin R.A., Fomina E.E., Ignatyev I.M. (2019). Method of determining disease severity in women with pelvic varicose veins. Angiol Sosud Khir.

[6] Sedaghat A.R. (2019). Understanding the minimal clinically important difference(MCID)of patient-reported outcome measures. Otolaryngol Head Neck Surg.

[7] Perrot S., Lantéri-Minet M. (2019). Patients' global impression of change in the management of peripheral neuropathic pain: clinical relevance and correlations in daily practice. Eur J Pain.

[8] Marcus J., Lasch K., Wan Y., Yang M., Hsu C., Merante D. (2018). An assessment of clinically important differences on the worst pain severity item of the modified brief pain inventory in patients with diabetic peripheral neuropathic pain. Pain Res Manag.

[9] Jayadevappa R., Cook R., Chhatre S. (2017). Minimal important difference to infer changes in health-related quality of life-a systematic review. J Clin Epidemiol.

[10] Marcelin C., Izaaryene J., Castelli M. (2017). Embolization of ovarian vein for pelvic congestion syndrome with ethylene vinyl alcohol copolymer (Onyx®). Diagn Interv Imaging.

[11] Maleux G., Stockx L., Wilms G., Marchal G. (2000). Ovarian vein embolization for the treatment of pelvic congestion syndrome: long-term technical and clinical results. J Vasc Interv Radiol.

[12] Sozutok S., Piskin F.C., Balli H.T., Onan H.B., Kaya O., Aksungur E.H. (2022). Efficacy of the endovascular ovarian vein embolization technique in pelvic venous congestion syndrome. Pol J Radiol.

[13] Jones I.A., Togashi R., Heckmann N., Vangsness C.T. (2020). Minimal clinically important difference (MCID) for patient-reported shoulder outcomes. J Shoulder Elbow Surg.

[14] Gavrilov S.G., Karalkin A.V., Mishakina N.Y., Grishenkova A.S. (2023). Hemodynamic and neurobiological factors for the development of chronic pelvic pain in patients with pelvic venous disorder. J Vasc Surg Venous Lymphat Disord.

[15] Bałabuszek K., Toborek M., Pietura R. (2022). Comprehensive overview of the venous disorder known as pelvic congestion syndrome. Ann Med.

[16] Barge T.F., Uberoi R. (2022). Symptomatic pelvic venous insufficiency: a review of the current controversies in pathophysiology, diagnosis, and management. Clin Radiol.

[17] Assi I.Z., Lynch S.R., Ricker B.D. (2024). A comparative study of altered hemodynamics in iliac vein compression syndrome. Front Bioeng Biotechnol.

[18] Jaworucka-Kaczorowska A., Simka M. (2025). Anatomical, pathophysiological, and clinical aspects of extra-pelvic varicose veins of pelvic origin. Diagnostics (Basel).

[19] Lakhanpal G., Kennedy R., Lakhanpal S., Sulakvelidze L., Pappas P.J. (2021). Pelvic venous insufficiency secondary to iliac vein stenosis and ovarian vein reflux treated with iliac vein stenting alone. J Vasc Surg Venous Lymphat Disord.

[20] Pennec V.L., Douane F., Brun J.L. (2025). Endovascular management of pelvic congestion syndrome: an expert consensus Statement from the French Society of Cardiovascular Imaging (SFICV), Interventional Radiology Federation (FRI), College of French Radiology Teachers (CERF), and French Society of Women’s Imaging (SIFEM). Diagn Interv Imaging.

[21] Jambon E., Le Bras Y., Petitpierre F. (2020). MRI associated factors of clinical efficacy of embolization in patients with pelvic venous insufficiency. Diagn Interv Imaging.

